# Global Variability in the Implementation of Gastric Cancer Prevention Guidelines: A Multinational Survey

**DOI:** 10.1055/a-2915-9599

**Published:** 2026-07-31

**Authors:** Apostolis Papaefthymiou, Giulio Antonelli, Marcello Maida, Renata Nobre, Stefano Baraldo, Dimitrije Damjanov, Hugo Uchima, Zuzana Vackova, Sunil Gupta, Tony He, Helena Degroote, Lynn Debels, Tolga Düzenli, Mario Tadic, Theodor Voiosu, Alessandro Rimondi, Djilani Bouberra, Karim Hamesch, Asmaa Gameel, Enrik Aguilla, Michael Mwachiro, Yuki Okubo, Stavros Dimitriadis, Katarzyna M. Pawlak, Ibrahim Mostafa, on behalf of ESGE (EYE) Committee and WEO Emerging Stars Program, ESGE Young Endoscopists (EYE) Committee:, G Antonelli, D Damjanov, L Debels, M Díaz--Alcázar, S Dimitriadis, K Hamesch, T He, G Olsen, A Papaefthymiou, E Santos-Perez, Z Vacková, K Pawlak, World Endoscopy Organization (WEO) Emerging Stars Program:, M Maida, A Papaefthymiou, S Gupta, T Voiosu, S Baraldo, P Pal, M Mwachiro, T Zuchelli, H Uchima, E Aguila, D Bouberra, H Degroote, T Düzenli, A Gameel, T Khurelbaatar, S Lakkasani, H Maulahela, R Nobre, Y Okubo, A Rimondi, A Taiymi, I Mostafa

**Affiliations:** 1 Faculty of Medicine 37786 University of ThessalyVolosGreece; 2 Department of GastroenterologyUniversity Hospital of LarissaLarissaGreece; 3 Gastroenterology and Digestive Endoscopy Unit 638740 Ospedale dei CastelliRomeItaly; 4 Department of Medicine and Surgery 217140 Kore University of EnnaEnnaItaly; 5 Department of Gastroenterology 215027 Universidade de Sao Paulo Instituto do Cancer do Estado de Sao PauloSão PauloSPBrazil; 6 Department of Gastroenterology 37884 Universidade de São Paulo Faculdade de MedicinaSão PauloSPBrazil; 7 Faculty of Medicine 84981 University of Novi SadNovi SadVojvodinaSerbia; 8 Clinic for Gastroenterology and HepatologyUniversity Clinical Center of VojvodinaNovi SadVojvodinaSerbia; 9 Hospital Universitario Germans Trias i Pujol; Centro de Investigación Biomédica en Red de Enfermedades Hepáticas y DigestivasBadalonaCommunity of MadridSpain; 10 Department of Gastrointestinal Endoscopy; Department of Medicine, 1st Faculty of MedicineMilitary University Hospital Prague; Charles UniversityPraguePragueCzech Republic; 11 Division of Gastroenterology 10071 St Michael’s HospitalTorontoCanada; 12 Department of Gastroenterology and Hepatology 70493 Vrije Universiteit Brussel, Universitair Ziekenhuis BrusselBrusselsBrusselsBelgium; 13 Department of Gastroenterology and Hepatology 60201 University Hospital BrusselsBrusselsBelgium; 14 Gastroenterology 485536 Hitit University Faculty of MedicineÇorumÇorum ProvinceTurkey; 15 Department of GastroenterologyUniversity Hospital Dubrava, Zagreb, Croatia; Faculty of Pharmacy and Biochemistry, University of ZagrebZagrebCroatia; 16 Department of GastroenterologyColentina Clinical Hospital, Carol Davila Faculty of MedicineBucharestRomania; 17 Royal Free Unit for Endoscopy 4965 Royal Free London NHS Foundation TrustLondonUnited Kingdom of Great Britain and Northern Ireland; 18 Gastroenterology UnitCentre Anti Cancer de BatnaBatnaBatna ProvinceAlgeria; 19 Medical Clinic III, Gastroenterology, Metabolic Diseases and Intensive CareUniversity Hospital RWTH AachenAachenGermany; 20 Interdisciplinary EndoscopyUniversity Hospital RWTH AachenAachenGermany; 21 Department of Gastroenterology and Hepatology 68780 Mansoura University Faculty of MedicineMansouraDakahlia GovernorateEgypt; 22 Institute of Digestive and Liver DiseasesSt. Luke’s Medical Center Global City, Taguig, Philippines; School of Medicine and Public Health, Ateneode Manila UniversityTaguigPhilippines; 23 Surgery and GastroenterologyAvenue HealthCareBometRiftvalleyKenya; 24 Department of Gastrointestinal Oncology 53312 Osaka International Cancer InstituteOsakaOsakaJapan; 25 Private PracticeThessalonikiGreece; 26 Endoscopy UnitHospital of the Ministry of Interior and AdministrationSzczecinPoland; 27 Department of Gastroenterology and HepatologyTheodor Bilharz Research InstituteCairoEgypt

**Keywords:** endoscopy upper GI tract, precancerous conditions & cancerous lesions (dysplasia and cancer) stomach, diagnosis and imaging (inc chromoendoscopy, NBI, iSCAN, FICE, CLE), quality and logistical aspects, training, image and data processing, documentation

## Abstract

**Background and study aim** Gastric cancer (GC) remains a major cause of cancer-related mortality worldwide. Although international guidelines recommend strategies for screening, diagnosis, and surveillance of gastric premalignant conditions, their implementation in clinical practice remains uncertain. This study aimed to evaluate global endoscopic practices and adherence to GC prevention guidelines.

**Patients and methods** We conducted a multinational web-based cross-sectional survey between August and September 2025 among endoscopists from 32 countries. A 28-item questionnaire assessed demographics, screening strategies, endoscopic technical practices, pathology infrastructure, *H. pylori* management, and surveillance policies. The primary outcome was characterization of current practices in GC screening and management of premalignant gastric conditions. Secondary outcomes included variability according to country and experience, and adherence to guidelines. Descriptive statistics, chi-square testing, and the Index of Qualitative Variation assessed heterogeneity. A composite guideline adherence score based on six indicators was calculated, and hierarchical clustering identified country-level adherence patterns.

**Results** A total of 863 physicians participated, most of whom were certified gastroenterologists. Routine *H. pylori* testing during endoscopy was reported by 73.7% ( *n* = 635) of respondents, while access to high-definition endoscopy with virtual chromoendoscopy was available to 58.7% ( *n* = 507). Although photo-documentation was widely available (90.4%, *n* = 780), only 40.6% ( *n* = 350) consistently documented recommended anatomical sites and 20.4% ( *n* = 176) used structured reporting systems. Guideline-based surveillance was reported by 85.7% ( *n* = 737) of respondents. Cluster analysis identified three country-level adherence groups with mean scores of approximately 0.83, 0.60, and 0.41.

**Conclusions** Implementation of GC prevention guidelines varies worldwide. Infrastructure, training, and healthcare organization appear to be key determinants of adherence rather than national GC incidence.

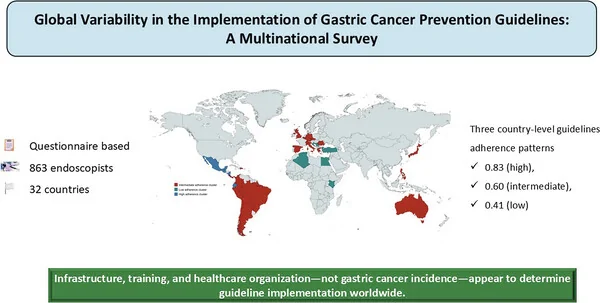

## Introduction

Gastric cancer (GC) remains one of the leading causes of cancer-related mortality worldwide, despite a gradual decline in incidence in several Western countries. 1 2 Marked geographic variation persists, with the highest age-standardized incidence rates (ASR) observed in East Asia and parts of Latin America, while lower rates are reported in Northern Europe and North America. 1 2 3

Organized screening programs have been implemented in high-incidence countries, whereas most Western countries rely on opportunistic detection and risk-based surveillance. International societies have issued recommendations for the management of gastric precancerous conditions, including technical guidance and information about diagnosis, treatment, and surveillance. 4 5 6 7 8 9 10 However, the extent to which these recommendations are implemented in daily clinical practice across different healthcare systems remains unclear. A recent European multicenter retrospective study across seven countries demonstrated that, although adherence has been improved for the majority of guideline recommendations, substantial gaps remained, highlighting that guideline adherence varies widely across practices. 11

Substantial variability may exist in access to high-definition (HD) endoscopy, virtual chromoendoscopy (VCE), expert gastrointestinal (GI) pathologists, and advanced resection techniques such as endoscopic submucosal dissection (ESD). Moreover, real-world approaches to *H. pylori* testing and eradication during endoscopy have not been systematically assessed on a global scale.

The aim of this international survey was to evaluate current practices in the screening, diagnosis, and surveillance of GC and premalignant gastric conditions among endoscopists.

## Methods

### Study Design and Participants

This was a multinational, web-based cross-sectional survey conducted between August and September 2025. The questionnaire was distributed through members of the World Endoscopy Organization (WEO) Emerging Stars group and the European Society of Gastroenterology Young Endoscopists (EYE) Committee, who coordinated dissemination within their respective countries. Participation was voluntary and anonymous and physicians from 32 countries across Europe, the Americas, Africa, Asia, and Australia completed the survey.

As the study did not involve patient-identifiable data or any interventions, IRB approval was not required.

### Questionnaire Development

A 28-item questionnaire ( **Supplementary file 1** ) was developed through investigator consensus and structured into four domains:

- Demographics and professional status
- Screening strategies and endoscopic quality practices
- Histological sampling and pathology infrastructure
- *Helicobacter pylori* management and surveillance policies

Items 1–6 captured demographic and professional characteristics together with the local availability of a GC screening program, while the remaining items mapped on to the three clinical domains of screening strategies and endoscopic quality practices, histological sampling and pathology infrastructure, and *H. pylori* management and surveillance policies. Each item was derived from a specific recommendation of current international guidelines 4 5 6 7 8 9 10 to ensure that the instrument reflected the key steps of guideline-concordant care; a complete item-by-item mapping to the respective domains, together with the guideline basis and rationale for each item, is provided in Supplementary file 2.

The questionnaire was developed by a multinational group drawn from the ESGE Young Endoscopists (EYE) Committee and the WEO Emerging Stars Program. An initial pool of candidate items was generated from the recommendations of current international guidelines on the management of gastric premalignant conditions 4 5 6 7 8 9 10 and was reviewed by the group, with regard to clinical relevance, clarity, and nonredundancy. The questionnaire was originally drafted in English and manually translated into native languages, where necessary. Online consensus meetings were conducted to ensure conceptual equivalence across translations.

The final survey was implemented using a secure Google Forms platform to share with the endoscopists all over the world and facilitate data collection.

### Outcomes

The primary outcome of this study was to record current practices of endoscopists in GC screening and premalignant conditions management.

Secondary outcomes included variability between countries, working environments, endoscopist experience, and compliance to existing guidelines. Potential differences according to the ASR were also evaluated; however, due to the low numbers of participants from countries with intermediate (10–20 per 100,000) and high (>20 per 100,000) risk of GC, we elected to merge these subgroups and use a limit of 10 per 100,000 to differentiate low- from higher-risk regions.

### Statistical Analysis

Continuous variables were expressed as mean ± standard deviation (SD), while categorical variables were reported as absolute frequencies and percentages. Comparisons between categorical variables were performed using the chi-square test. A two-sided *p* -value < 0.05 was considered to indicate statistical significance. Heterogeneity across independent variables was assessed using the Index of Qualitative Variation (IQV), with values ranging from 0, indicating no diversity, to 1, indicating maximum heterogeneity.

A composite guideline adherence score was derived by calculating the mean of six equally weighted binary quality indicators, resulting in a continuous score ranging from 0 to 1 **(Supplementary file 3)** . The six indicators were selected to reflect core recommendations that are concordant across the major international guidelines on the management of gastric premalignant conditions (ESGE MAPS III, AGA, ACG, BSG, and APAGE), 4 5 6 7 8 9 10 with the ESGE MAPS III guideline used as the primary reference standard; because these indicators capture recommendations common to all of these documents, adherence could be assessed uniformly across countries despite differences between individual national or regional guidelines. Several additional questionnaire items addressed the broader continuum of GC prevention beyond the three core domains—including chemoprevention (aspirin use), risk-based patient selection (age cut-offs), and access to therapeutic resources (ESD)—and were analyzed descriptively to characterize real-world practice; these contextual items were not incorporated into the composite adherence score, which was restricted to the six core quality indicators. Mean country-level adherence scores were subsequently calculated and standardized as z-scores prior to cluster analysis. Hierarchical clustering was then performed to identify patterns of guideline adherence across countries, using Ward’s minimum variance method and squared Euclidean distance as the similarity measure. The optimal number of clusters was determined on the basis of dendrogram inspection and evaluation of agglomeration coefficients. All statistical analyses were conducted using SPSS Statistics, version 31.0 (IBM Corp., Chicago, IL, USA).

## Results

In all, 863 physicians from 32 countries participated in this questionnaire-based survey. This included 94 (10.9%) participants from countries with the highest ASR (≥10 per 100,000 population is recorded in Japan, Turkey, and some Latin American countries), and 769 (89.1%) participants with the lowest ASR. 3

### Participant Characteristics

Among 863 respondents, the majority were certified gastroenterologists (84.3%, *n* = 728), with 26.3% ( *n* = 230) reporting some kind of dedicated training in upper gastrointestinal (UGI) diseases and advanced endoscopic assessment of premalignant gastric conditions. The highest proportions of this subgroup were from Costa Rica (66.7%), Philippines (60%), Brazil (59.6%), the UK (50%), Japan (50%), Peru (40%), Spain (38.5%), Argentina (35.7%), and Australia (30%). Over half (50.9%, *n* = 439) had more than 10 years of UGI endoscopy experience. Participants worked in public hospitals (45.4%, *n* = 392), private practice (29.6%, *n* = 255), or academic institutions (25%, *n* = 216). Table 1 summarizes the main characteristics of the participants per country.

**Table 1:** The main characteristics of the participants per country

		Belgium	UK	Germany	Philippines	Switzerland	Spain	Kenya	Croatia	Italy	Greece	Argentina	Japan	Algeria	Egypt	Brazil	Chech Republic	Serbia	Australia	Romania	Bolivia	Chile	Colombia	Costa Rica	Dominican Republic	Ecuador	Guatemala	Mexico	Nicaragua	Panama	Paraguay	Peru	Turkey
ASR		<10	<10	<10	<10	<10	<10	<10	<10	<10	<10	<10	<10	<10	<10	<10	<10	<10	<10	<10	<10	≥10	≥10	≥10	<10	≥10	<10	<10	<10	≥10	<10	≥10	≥10
Age	<30	1	0	0	0	0	3	0	5	0	4	1	0	2	0	4	3	3	1	3	0	0	0	0	0	0	0	0	0	0	0	0	0
		3,3%	0,0%	0,0%	0,0%	0,0%	10,0%	0,0%	16,7%	0,0%	13,3%	3,3%	0,0%	6,7%	0,0%	13,3%	10,0%	10,0%	3,3%	10,0%	0,0%	0,0%	0,0%	0,0%	0,0%	0,0%	0,0%	0,0%	0,0%	0,0%	0,0%	0,0%	0,0%
	30-45	27	10	9	5	1	37	3	19	20	67	16	4	23	6	57	27	41	45	11	2	0	3	10	0	0	0	1	0	0	0	6	36
		5,6%	2,1%	1,9%	1,0%	0,2%	7,6%	0,6%	3,9%	4,1%	13,8%	3,3%	0,8%	4,7%	1,2%	11,7%	5,6%	8,4%	9,3%	2,3%	0,4%	0,0%	0,6%	2,1%	0,0%	0,0%	0,0%	0,2%	0,0%	0,0%	0,0%	1,2%	7,4%
	46-60	15	1	0	0	0	9	0	6	1	79	23	0	3	0	36	15	37	1	3	1	1	7	2	0	0	1	0	1	1	1	7	7
		5,8%	0,4%	0,0%	0,0%	0,0%	3,5%	0,0%	2,3%	0,4%	30,6%	8,9%	0,0%	1,2%	0,0%	14,0%	5,8%	14,3%	0,4%	1,2%	0,4%	0,4%	2,7%	0,8%	0,0%	0,0%	0,4%	0,0%	0,4%	0,4%	0,4%	2,7%	2,7%
	>60	5	1	0	0	0	3	1	0	1	30	2	0	1	0	12	6	12	3	0	0	2	5	0	1	1	0	1	0	0	0	2	0
		5,6%	1,1%	0,0%	0,0%	0,0%	3,4%	1,1%	0,0%	1,1%	33,7%	2,2%	0,0%	1,1%	0,0%	13,5%	6,7%	13,5%	3,4%	0,0%	0,0%	2,2%	5,6%	0,0%	1,1%	1,1%	0,0%	1,1%	0,0%	0,0%	0,0%	2,2%	0,0%
Professional status	Supervised trainee	1	0	0	0	0	3	0	7	0	12	1	0	1	0	3	3	9	4	2	0	0	0	0	0	0	0	0	0	0	0	0	2
		2,1%	0,0%	0,0%	0,0%	0,0%	6,3%	0,0%	14,6%	0,0%	25,0%	2,1%	0,0%	2,1%	0,0%	6,3%	6,3%	18,8%	8,3%	4,2%	0,0%	0,0%	0,0%	0,0%	0,0%	0,0%	0,0%	0,0%	0,0%	0,0%	0,0%	0,0%	4,2%
	Trainee independent in EGD	2	1	4	0	0	0	0	6	1	9	0	0	0	0	0	8	21	12	7	0	0	0	0	0	0	0	0	0	0	0	0	4
		2,7%	1,3%	5,3%	0,0%	0,0%	0,0%	0,0%	8,0%	1,3%	12,0%	0,0%	0,0%	0,0%	0,0%	0,0%	10,7%	28,0%	16,0%	9,3%	0,0%	0,0%	0,0%	0,0%	0,0%	0,0%	0,0%	0,0%	0,0%	0,0%	0,0%	0,0%	5,3%
	Nurse endoscopist	1	0	0	0	0	0	0	0	1	0	1	0	6	1	1	0	0	0	0	0	0	0	0	0	0	0	0	0	0	0	0	1
		8,3%	0,0%	0,0%	0,0%	0,0%	0,0%	0,0%	0,0%	8,3%	0,0%	8,3%	0,0%	50,0%	8,3%	8,3%	0,0%	0,0%	0,0%	0,0%	0,0%	0,0%	0,0%	0,0%	0,0%	0,0%	0,0%	0,0%	0,0%	0,0%	0,0%	0,0%	8,3%
	Gastroenterologist	26	6	5	3	0	24	2	12	14	129	20	3	14	1	92	30	38	22	8	1	2	8	7	0	0	1	0	0	0	0	6	27
		5,2%	1,2%	1,0%	0,6%	0,0%	4,8%	0,4%	2,4%	2,8%	25,7%	4,0%	0,6%	2,8%	0,2%	18,4%	6,0%	7,6%	4,4%	1,6%	0,2%	0,4%	1,6%	1,4%	0,0%	0,0%	0,2%	0,0%	0,0%	0,0%	0,0%	1,2%	5,4%
	Expert in UGI	18	5	0	2	1	25	2	5	6	30	20	1	8	4	13	10	25	12	0	2	1	7	5	1	1	0	2	1	1	1	9	9
		7,9%	2,2%	0,0%	0,9%	0,4%	11,0%	0,9%	2,2%	2,6%	13,2%	8,8%	0,4%	3,5%	1,8%	5,7%	4,4%	11,0%	5,3%	0,0%	0,9%	0,4%	3,1%	2,2%	0,4%	0,4%	0,0%	0,9%	0,4%	0,4%	0,4%	4,0%	4,0%
Current work	Public	28	4	4	0	1	27	0	24	16	56	13	0	10	0	63	21	47	33	10	1	2	5	10	1	1	0	1	0	1	0	8	5
		7,1%	1,0%	1,0%	0,0%	0,3%	6,9%	0,0%	6,1%	4,1%	14,3%	3,3%	0,0%	2,6%	0,0%	16,1%	5,4%	12,0%	8,4%	2,6%	0,3%	0,5%	1,3%	2,6%	0,3%	0,3%	0,0%	0,3%	0,0%	0,3%	0,0%	2,0%	1,3%
	Private	8	1	1	3	0	10	4	0	0	98	26	0	15	0	29	14	8	9	3	2	1	8	0	0	0	1	1	0	0	1	5	7
		3,1%	0,4%	0,4%	1,2%	0,0%	3,9%	1,6%	0,0%	0,0%	38,4%	10,2%	0,0%	5,9%	0,0%	11,4%	5,5%	3,1%	3,5%	1,2%	0,8%	0,4%	3,1%	0,0%	0,0%	0,0%	0,4%	0,4%	0,0%	0,0%	0,4%	2,0%	2,7%
	Academic	12	7	4	2	0	15	0	6	6	26	3	4	4	6	17	16	38	8	4	0	0	2	2	0	0	0	0	1	0	0	2	31
		5,6%	3,2%	1,9%	0,9%	0,0%	6,9%	0,0%	2,8%	2,8%	12,0%	1,4%	1,9%	1,9%	2,8%	7,9%	7,4%	17,6%	3,7%	1,9%	0,0%	0,0%	0,9%	0,9%	0,0%	0,0%	0,0%	0,0%	0,5%	0,0%	0,0%	0,9%	14,4%
Experience in UGI endoscopy	<5 years	5	1	4	2	0	8	2	16	7	23	5	0	11	2	22	10	31	21	11	0	0	2	4	0	0	0	1	0	0	0	0	17
		2,4%	0,5%	2,0%	1,0%	0,0%	3,9%	1,0%	7,8%	3,4%	11,2%	2,4%	0,0%	5,4%	1,0%	10,7%	4,9%	15,1%	10,2%	5,4%	0,0%	0,0%	1,0%	2,0%	0,0%	0,0%	0,0%	0,5%	0,0%	0,0%	0,0%	0,0%	8,3%
	5-10 years	17	6	1	3	1	16	0	3	9	43	6	3	10	1	22	13	14	24	2	2	0	2	3	0	0	0	0	0	0	0	5	13
		7,8%	2,7%	0,5%	1,4%	0,5%	7,3%	0,0%	1,4%	4,1%	19,6%	2,7%	1,4%	4,6%	0,5%	10,0%	5,9%	6,4%	11,0%	0,9%	0,9%	0,0%	0,9%	1,4%	0,0%	0,0%	0,0%	0,0%	0,0%	0,0%	0,0%	2,3%	5,9%
	>10 years	26	5	4	0	0	28	2	11	6	114	31	1	8	3	65	28	48	5	4	1	3	11	5	1	1	1	1	1	1	1	10	13
		5,9%	1,1%	0,9%	0,0%	0,0%	6,4%	0,5%	2,5%	1,4%	26,0%	7,1%	0,2%	1,8%	0,7%	14,8%	6,4%	10,9%	1,1%	0,9%	0,2%	0,7%	2,5%	1,1%	0,2%	0,2%	0,2%	0,2%	0,2%	0,2%	0,2%	2,3%	3,0%
Formal training in VCE and MAPS	Yes	14	6	1	3	0	20	1	2	6	21	15	2	3	0	65	10	11	15	3	2	2	3	8	1	1	1	2	1	1	0	6	4
		6,1%	2,6%	0,4%	1,3%	0,0%	8,7%	0,4%	0,9%	2,6%	9,1%	6,5%	0,9%	1,3%	0,0%	28,3%	4,3%	4,8%	6,5%	1,3%	0,9%	0,9%	1,3%	3,5%	0,4%	0,4%	0,4%	0,9%	0,4%	0,4%	0,0%	2,6%	1,7%
	No	34	6	8	2	1	32	3	28	16	159	27	2	26	6	44	41	82	35	13	1	1	12	4	0	0	0	0	0	0	1	9	39
		5,4%	0,9%	1,3%	0,3%	0,2%	5,1%	0,5%	4,4%	2,5%	25,2%	4,3%	0,3%	4,1%	0,9%	7,0%	6,5%	13,0%	5,5%	2,1%	0,2%	0,2%	1,9%	0,6%	0,0%	0,0%	0,0%	0,0%	0,0%	0,0%	0,2%	1,4%	6,2%

### Screening Strategies

Organized GC screening programs were reported primarily in areas with high-incidence of GC, including Japan and several Latin American nations (Chile, Costa Rica, and Ecuador). It should be noted, however, that these figures reflect respondents’ perceptions of locally available programs; among the surveyed countries, only Japan operates a formally established nationwide population-based screening program, whereas the programs reported elsewhere are largely regional, opportunistic, or emerging rather than organized population-based initiatives. However, adherence to screening programs was variable, with substantial within-country heterogeneity observed (IQV: 0.67). Furthermore, 15.6% of participants from Brazil, 15.1% from Serbia, and 17.6% from Romania were not aware whether a screening program existed in their respective countries.

Only one-third of respondents applied a defined age cut-off for screening or surveillance endoscopy, with higher rates in Asia and high-ASR countries. Screening EGD was most commonly performed only when another clinical indication was present (63.8%, *n* = 551), whereas 4.6% ( *n* = 40) answered that they perform EGD as per patient request, and 10% ( *n* = 86) every time with screening colonoscopy. Opportunistic screening during colonoscopy was more frequent in the private sector (16.1%). Notably, 81.1% ( *n* = 700) reported systematic gastric assessment during EGD performed for other indications.

### Endoscopic Quality and Technical Practice

Patient preparation beyond fasting was not routinely recommended by 71.8% ( *n* = 619) of respondents. Mucolytic agents were used by 26.9% ( *n* = 232), with higher utilization among experienced and UGI-focused endoscopists (29.9% and 26.1%, respectively). The highest rate of mucolytic agent use was recorded in Brazil (84.4%).

**Fig. 1:**
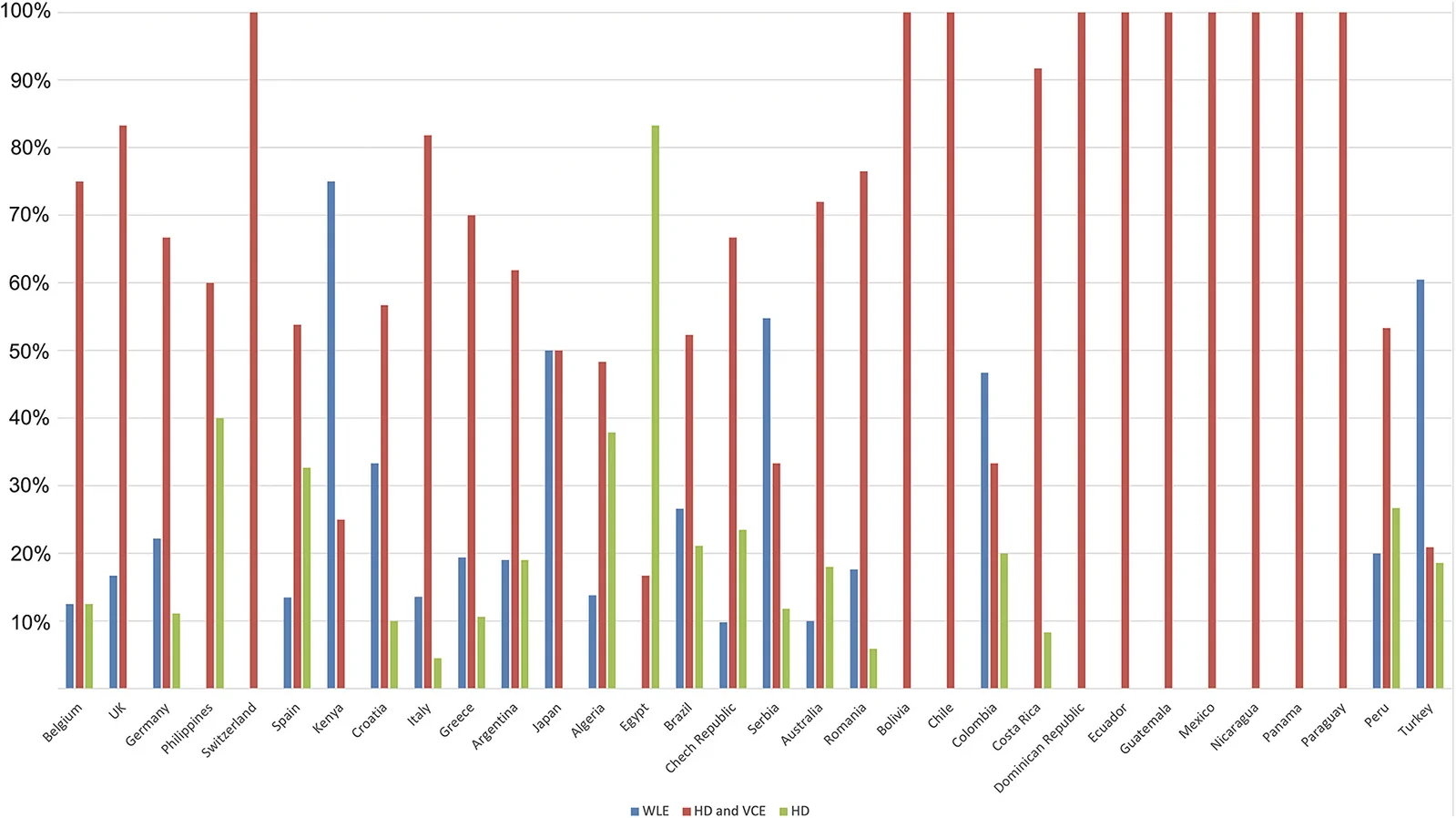
Bar chart describing the access to various types of endoscopes (WLE, white light endoscopes; HD and VCE, high-definition and virtual chromoendoscopy; HD, high-definition endoscopy) in each country.

Although 58.7% ( *n* = 507) reported access to high-definition endoscopy with virtual chromoendoscopy (HD-VCE), marked variability in its use across countries was identified (IQV up to 0.92), with the highest diversity observed in Argentina, Algeria, Colombia, and Brazil. Older-generation endoscopes, non-HD and without virtual chromoendoscopy, remained common in several regions, including Kenya (75%), Turkey (60.5%), Serbia (54.8%), and Colombia (46.5%). The distribution of endoscope type differed significantly across work settings ( *p* < 0.05). In the private practice, the use of HD-VCE was higher compared with the academic and public sectors. Fig. 1 illustrates the distribution of different equipment among countries.

Photo-documentation was widely available (90.4%, *n* = 780), yet only 40.6% ( *n* = 350) consistently documented all recommended anatomical sites. Importantly, only 20.4% ( *n* = 176) used structured endoscopic reporting systems with validated scales for gastric atrophy and intestinal metaplasia. Use of validated EGD reporting scales differed significantly according to professional status, with experts in UGI endoscopy reporting validated scales more frequently than all other groups (p<0.05), whereas no statistically significant differences were observed in the EGD reporting practices across public, private, and academic sectors.

Regarding tissue sampling for chronic atrophic gastritis, the Sydney protocol was followed by 92.2% ( *n* = 796) of respondents, although modifications were common. More specifically, 57.5% ( *n* = 458) adjusted this protocol by adding samples from the incisura and 26.4% ( *n* = 210) took additional targeted biopsies when there was a visible lesion. However, 10% ( *n* = 87) placed biopsy specimens in a single container rather than separate jars. This approach was common not only in cases of nurse endoscopists (16.7%) but also with experts (12.3%). Less than half (48.1%, *n* = 415) reported consistent access to an expert gastrointestinal pathologist, while 27.8% ( *n* = 240) do not have expert pathologist at all. Significant disparities were observed across countries, highlighting infrastructure inequalities.

### *Helicobacter pylori* Management

Routine *H. pylori* testing during EGD was performed by 73.7% ( *n* = 635) of respondents regardless of the indication for EGD, primarily via histology (59.4%, *n* = 513). Testing rates increased to 88.2% in the suspicion of intestinal metaplasia. Although intestinal metaplasia represents an advanced stage at which *H. pylori* may no longer be detectable on histology, current guidelines still recommend testing for and eradicating the infection in this setting, which is consistent with the higher testing rate observed. The countries with the highest rates of omitting *H. pylori* investigation were Germany, the UK, Spain, Croatia, and Czech Republic. The approach to *H. pylori* testing during EGD differed significantly across professional status groups ( *p* < 0.05).

### Surveillance Strategy

Adherence to guideline-based surveillance for premalignant conditions was high overall (85.7%, *n* = 737), although reliance on individualized intervals remained notable in certain regions. This is more commonly observed among participants from Kenya (50%), Algeria (37.9%), Germany (33.3%), Egypt (33.3%), and Croatia (30.0%). In case of GC diagnosis, the majority of participants recommended first degree relatives to undergo *H. pylori* testing with (66.3%) or without (13.1%) EGD, while 20.5% did not follow a specific plan.

Access to ESD was reported by 81.2% ( *n* = 701) overall but showed pronounced geographic heterogeneity ( *p* < 0.001). Countries such as the UK, Germany, and Japan demonstrated near-universal access with minimal variability (IQV = 0.00–0.08). In contrast, marked heterogeneity (IQV = 0.97–1.00), due to higher rates of unavailability, was observed in Kenya (50%), Algeria (58.6%), Egypt (33.3%), Serbia (33.3%), Argentina (28.6%), and Greece (27.2%).

### Compliance to Guidelines

**Fig. 2:**
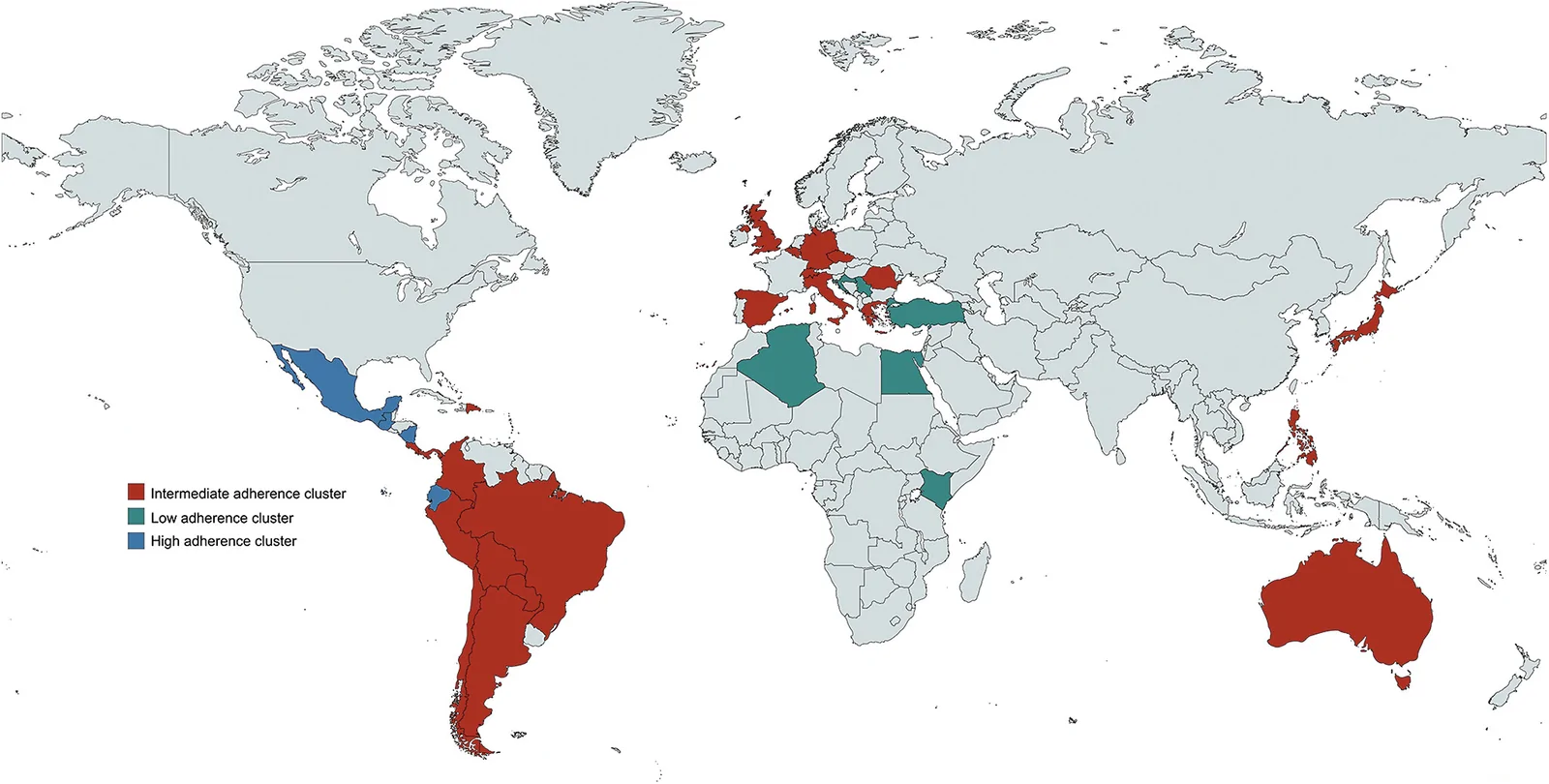
Global map illustrating the different adherence clusters. Green color corresponds to low adherence, red to intermediate adherence, and blue to high adherence.

Countries were grouped into three adherence patterns according to the analysis based on six indicators ( Table 2 ). A high-adherence cluster (Ecuador, Guatemala, Mexico, Nicaragua) demonstrated the highest composite scores (mean ≈ 0.83); however, limited number of endoscopists represented these countries. The intermediate-adherence cluster (majority of European and several Latin American countries) that demonstrated moderate composite scores (mean ≈ 0.60) included the largest part of our sample. These countries showed good availability of HD endoscopy and photo-documentation but inconsistent use of chromoendoscopy and structured reporting systems. A low-adherence cluster (Kenya, Algeria, Egypt, Serbia, Turkey, Croatia) exhibited the lowest composite scores (mean ≈ 0.41). This group was characterized by limited access to HD-VCE, low implementation of structured reporting systems, reduced chromoendoscopy utilization, and inconsistent surveillance strategies. Fig. 2 illustrates a global map of the participating countries grouped in these clusters.

**Table 2:** Country-based adherence clusters according to the composite guideline adherence model.

Cluster	Countries included	Mean adherence score	Key characteristics
High adherence	Ecuador, Guatemala, Mexico, Nicaragua	≈0.83	High availability of HD-VCE, frequent use of structured reporting systems, high adherence to biopsy protocols and surveillance recommendations
Intermediate adherence	Majority of European countries and several Latin American countries	≈0.60	Good availability of HD endoscopy and photo-documentation but inconsistent use of chromoendoscopy, structured reporting systems, and standardized surveillance strategies
Low adherence	Kenya, Algeria, Egypt, Serbia, Turkey, Croatia	≈0.41	Limited access to HD-VCE, low implementation of structured reporting systems, reduced use of chromoendoscopy, limited pathology infrastructure, and inconsistent surveillance practices

No significant association was observed between GC incidence category (ASR ≥ 10 vs <10) and country-level adherence clusters (χ ^2^ = 0.28, *p* = 0.868).

## Discussion

This multinational survey provides a comprehensive and contemporary overview of real-world practices in GC screening and management of premalignant conditions across 32 countries. Although overall self-reported adherence to surveillance recommendations was high (85.7%) and most respondents were experienced gastroenterologists, our findings reveal substantial heterogeneity in infrastructure, technique, and implementation of structured diagnostic strategies. A major novel finding of our study is the identification of three distinct country-level adherence phenotypes, yet these were not associated with local GC incidence (ASR ≥10 vs. <10; *p* = 0.868), suggesting that disease burden alone does not determine quality of care. Rather, infrastructure availability, training exposure, and system organization appear to be the primary drivers of compliance.

Our findings are consistent with a recent study demonstrating a persistent implementation gap despite gradual improvements over time. 11 In that study, VCE utilization increased significantly over a decade, and biopsy protocol adherence improved in most centers; however, structured staging systems and surveillance documentation remained suboptimal in many institutions. 11 Similarly, in our study, although 58.7% of participants reported access to HD-VCE, its consistent use was far from universal, and only 20.4% reported use of structured reporting systems with validated scoring systems, like the Kimura–Takemoto scale. 4 12 Another interesting finding was that only 40.6% consistently photo-documented all recommended anatomical sites, highlighting a critical gap in quality standardization. These findings mirror the European experience, where improved technical availability did not automatically translate into systematic implementation. 11

The variability observed in our study is further supported by Madhu et al., 13 where adherence to best practices for detection of premalignant UGI lesions was below predefined thresholds in most domains. Only 58.5% of endoscopists had received formal training in detection of premalignant lesions, and appropriate image-enhanced endoscopy was available to only 58%, a finding that is remarkably similar to our global HD-VCE access rate (58.7%). Both datasets emphasize that training and infrastructure limitations represent key barriers to optimal detection strategies. Additionally, in our study, the use of validated reporting systems was significantly higher among UGI-focused experts ( *p* < 0.05), reinforcing the critical role of targeted education.

Real-world deficiencies are not only confined to equipment or reporting. A retrospective analysis from the Netherlands and the UK showed that endoscopic recognition rates of gastric atrophy and intestinal metaplasia were only 48.5% and 16.3%, respectively, and adequate surveillance was performed in only 54.3% of patients. 14 These data closely align with our observation that, although 85.7% reported guideline-based surveillance, individualized or nonstandardized intervals remained common in several countries. Together, these studies highlight that even in low-incidence countries with advanced healthcare systems, awareness and systematic application of guidelines remain incomplete.

Screening strategies also revealed important discrepancies. Organized programs were largely confined to regions with high ASR such as Japan and selected Latin American countries, consistent with global epidemiologic patterns and current guidelines. 4 However, screening in most countries was opportunistic, frequently performed only when other indications were present (63.8%). Evidence from population-based studies underscores the importance of structured programs and adherence. Kim et al., 15 comparing national screening and opportunistic screening, demonstrated that compliance with screening intervals was more critical than the screening model itself, with regular follow-up associated with higher proportions of early GC detection (74% vs 53.8%). In contrast, irregular attendance was paradoxically associated with higher crude detection rates, likely reflecting symptomatic or higher-risk populations. These findings are particularly relevant to our data, where only one-third of respondents applied defined age cut-offs and opportunistic practices predominated outside organized programs. This evidence suggests that structured implementation and adherence monitoring determine screening effectiveness. A cluster randomized trial from China in a non-high-incidence rural area reported relatively low diagnostic yield (GC detection rate 0.55%) but acceptable early detection rates (33.3%) among screened individuals. 16 Interestingly, compliance was influenced by demographic and psychosocial factors. These findings resonate with our observation that high-incidence status alone was not associated with better adherence clusters. Structural and behavioral determinants—including education, awareness, reimbursement models, and culture—likely play a more decisive role than epidemiology per se.

Our cluster analysis provides an additional novel perspective by identifying three adherence phenotypes. The high-adherence cluster demonstrated composite scores around 0.83 but was represented by relatively small samples. The intermediate cluster (mean ≈ 0.60) comprised most European and Latin American countries, characterized by adequate availability of HD equipment but inconsistent structured implementation. The low-adherence cluster (mean ≈ 0.41) was marked by infrastructure limitations, low HD-VCE access, limited pathology support, and inconsistent surveillance practices. Importantly, this clustering was not explained by ASR category, reinforcing the notion that infrastructure constraints and lack of standardization are stronger determinants than cancer incidence. This has important global health implications, thereby investments in training, equipment renewal, pathology collaboration, and reporting standardization may yield greater improvements than incidence-driven policy alone. From a clinical standpoint, these phenotypes are not merely statistical groupings but correspond to differences in the care that a patient with premalignant gastric conditions is likely to receive. In low-adherence settings, the parameters are expected to translate into a higher risk of missed or under-staged lesions and inadequate follow-up, whereas high-adherence settings approximate the standard of care. Framing the results in this way allows targeted, resource-sensitive interventions to be matched to each context—for example, equipment renewal and pathology support in low-adherence settings, versus reinforcement of structured reporting and standardized surveillance intervals in intermediate-adherence settings.

This study has also several limitations that should be acknowledged. First, data were self-reported and may be subject to recall and social desirability bias. Second, country representation was unequal, and response rates could not be calculated, limiting generalizability. Third, dissemination through young endoscopist networks may have introduced self-selection bias and potentially overestimated adherence. Fourth, country-level clustering was based on 32 countries with unequal sample sizes, limiting stability of cluster estimates. Countries with small samples appeared artificially homogeneous, while larger samples exhibited greater heterogeneity. In particular, several countries contributed only a handful of respondents (as illustrated in Fig. 2 ), so the corresponding estimates and adherence clusters—most notably the high-adherence cluster, which comprised countries with very small samples—should be regarded as exploratory rather than as precise national benchmarks, and direct comparisons between individual countries should be interpreted with considerable caution. Additionally, no weighting according to national population size was applied. Finally, patient-level outcomes were not assessed; therefore, we cannot correlate reported practices with detection rates or clinical endpoints.

Despite these limitations, this study represents one of the largest international assessments of real-world practices on premalignant conditions management and GC prevention, revealing that the gap between guidelines and practice is a global phenomenon. Infrastructure disparities, limited structured reporting, inconsistent use of image-enhanced endoscopy, and variability in surveillance implementation remain key barriers.

In conclusion, this multinational survey demonstrates that while awareness of GC prevention guidelines is widespread, real-world implementation remains heterogeneous and strongly influenced by infrastructure, training, and system organization rather than national cancer incidence. Future strategies should prioritize structured quality indicators, formal training in advanced endoscopic assessment, standardized reporting systems, and resource-sensitive implementation frameworks. Bridging the gap between guidelines and practice will require not only technical upgrades but also sustained educational initiatives and health system–level commitment to quality assurance in GC prevention.

## Supplementary Material

Ergänzendes Material Supplementary Material
