# Supplementary material for: Global Variability in the Implementation of Gastric Cancer Prevention Guidelines: A Multinational Survey
Source: Endosc Int Open. 2026 Jul 31;14:a29159599. doi: 10.1055/a-2915-9599 (PMC13494750; doi:10.1055/a-2915-9599)
Supplement: Supplementary file 1 [file 10-1055-a-2915-9599_29164269.pdf]

Supplementary material

Global Variability in the Implementation of Gastric Cancer Prevention Guidelines: A Multinational Survey

Apostolis Papaefthymiou, Giulio Antonelli, Marcello Maida, Renata Nobre, Stefano Baraldo, Dimitrije Damjanov, Hugo Uchima, Zuzana Vackova, Sunil Gupta, Tony He, Helena Degroote, Lynn Debels, Tolga Düzenli, Mario Tadic, Theodor Voiosu, Alessandro Rimondi, Djilani Bouberra, Karim Hamesch, Asmaa Gameel, Enrik Aguila, Michael Mwachiro, Yuki Okubo, Stavros Dimitriadis, Katarzyna M Pawlak, Ibrahim Mostafa on behalf of ESGE EYE Committee and WEO Emerging Stars Program.

Supplementary Item 1. The 28-item questionnaire used in this study

Gastric cancer and premalignant lesions screening strategies among clinicians

Dear Colleague,

This is an anonymous questionnaire investigating the different policies and strategies on GC and PL screening among endoscopists in different countries. Please answer honestly in the below questions.

* Indicates required question

1. Country *

2. Age *

Mark only one oval.

- <30
- 30-45
- 46-60
- >60

3. What is your professional status? *

Mark only one oval.

- Trainee under supervision
- Trainee performing gastroscopy independently
- Nurse endoscopist
- Certified gastroenterologist
- Gastroenterologist expert in upper GI endoscopy/diseases

4. Where are you currently working? *

Mark only one oval.

- Public hospital
- Private sector
- Academic hospital

5. How many years are you performing upper GI endoscopy? *

Mark only one oval.

- < 5 years
- 5-10 years
- > 10 years

6. Is there a gastric cancer screening program in your country? *

Mark only one oval.

- Yes
- No
- Not sure

7. How often do you perform diagnostic gastroscopy (EGD) for gastric cancer/precancerous conditions screening? *

Mark only one oval.

- Never
- Every time that someone has screening colonoscopy
- Only if patient asks
- Only when there is another indication for gastroscopy
- According to the established screening program in my country

8. Aside from fasting do you routinely use any other preparation before diagnostic EGD? *

Mark only one oval.

- No
- A glass of water
- Mycolytic agents (e.g. simethicone)

9. When you perform gastroscopy for any indication, do you assess stomach for gastric cancer or precancerous conditions? *

Mark only one oval.

- Always
- Depending on the indication
- Occasionally

10. Do you use an age cut-off for GC screening?

Mark only one oval.

- Yes
- No

11. What type of endoscope do you use during diagnostic EGD? *

Mark only one oval.

- Conventional wight-light endoscopy (WLE)
- High-definition (HD) and virtual chromoendoscopy (VCE)
- HD-WLE

12. Do you have the infrastructure to provide photo-documentation? *

Mark only one oval.

- Yes, and I routinely use it
- Yes, but I do not use it
- No

13. In case that provide it, what are the normal areas of photo-documentation? (Please select all applicable choices)

Check all that apply.

- Proximal and mid esophagus
- Gastroesophageal junction
- Fundus
- Body
- Incisura
- Antrum
- Bulb
- Duodenum

14. Have you received dedicated, formal training in VCE and identification of premalignant gastric conditions? *

Mark only one oval.

- Yes
- No

15. Do you use chromoendoscopy (conventional or virtual) in every diagnostic EGD? *

Mark only one oval.

- Yes
- No

16. Do you test for H.pylori in every EGD? *

Mark only one oval.

- Yes, using the rapid urease test (CLO)
- Yes, with biopsies and histopathology
- No

17. When a patient needs chronic use of PPIs, do you test them for H.pylori? *

Mark only one oval.

- Yes
- No

18. Do you treat H.pylori in patients with established IM? *

Mark only one oval.

- Yes
- No

19. What is the biopsy pattern that you use when investigating for gastric atrophy (GA)/ intestinal metaplasia (IM)? *

Mark only one oval.

- Random
- Two from antrum/two from corpus
- Two from antrum/two from corpus/one or two from incisura
- Targeted biopsies only
- Targeted biopsies and mapping

Endosc Int Open | DOI 10.1055/a-2915-9599 | © The Author(s).

20. How do you treat biopsy specimens? *

Mark only one oval.

- All in one jar
- Separated depending on the existence of visible lesion and according to the sampling pattern

21. Once you diagnose someone with GC, what is your approach with first degree relatives? *

Mark only one oval.

- Nothing specific
- Guidance on Hp testing
- Guidance on Hp testing and EGD

22. How do you describe GA/IM in your EGD report? *

Mark only one oval.

- Using a validated scale (e.g. Kimura-Takemoto, EGGIM)
- Only descriptively

23. Do you have access to an expert GI pathologist, who provides detailed information on GA/IM? *

Mark only one oval.

- Yes
- No
- Occasionally

24. How do you plan your surveillance in patients with GA/IM? *

Mark only one oval.

- According to my experience
- Randomly
- According to national/regional guidelines

25. How often do you refer patients with suspicious lesions for a high-quality endoscopy? *

Mark only one oval.

- Never, due to unavailability of a specialized center
- Never, because I am an expert and have the equipment for a high-quality EGD
- Always

26. Do you prescribe aspirin in patients with premalignant gastric conditions? *

Mark only one oval.

- Always
- Only when there is CVS comorbidity after discussion with cardiologist
- Never

27. Do you follow-up patients with autoimmune gastritis with EGD for GC and carcinoid tumors? *

Mark only one oval.

- Yes, randomly
- Yes, every 3 years
- No

28. Do you have access to ESD locally or after referral to another center? *

Mark only one oval.

- Yes
- No

Supplementary file 2. Mapping of questionnaire items to study domains, with the corresponding international-guideline basis and rationale for inclusion.

No.	Questionnaire item	Domain
1	Country of practice	Demographics and professional status
2	Age of respondent	Demographics and professional status
3	Professional status (trainee, gastroenterologist, expert, nurse endoscopist)	Demographics and professional status
4	Current work setting (public, private, academic)	Demographics and professional status
5	Years performing upper GI endoscopy	Demographics and professional status
6	Existence of a gastric cancer screening programme in the country	Demographics and professional status
7	Frequency of diagnostic gastroscopy for screening of GC / precancerous conditions	Screening strategies and endoscopic quality practices
8	Additional mucosal preparation before EGD (beyond fasting)	Screening strategies and endoscopic quality practices
9	Assessment of the stomach for GC / precancerous conditions during EGD performed for any indication	Screening strategies and endoscopic quality practices
10	Use of an age cut-off for GC screening	Screening strategies and endoscopic quality practices
11	Type of endoscope used (WLE / HD-WLE / HD-VCE)	Screening strategies and endoscopic quality practices
12	Infrastructure to provide photo-documentation	Screening strategies and endoscopic quality practices
13	Anatomical sites routinely photo-documented	Screening strategies and endoscopic quality practices
14	Dedicated formal training in virtual chromoendoscopy and identification of premalignant conditions	Screening strategies and endoscopic quality practices
15	Routine use of (virtual) chromoendoscopy at every EGD	Screening strategies and endoscopic quality practices
16	Testing for *H. pylori* at every EGD	*H. pylori* management and surveillance policies
17	Testing for *H. pylori* before long-term PPI therapy	*H. pylori* management and surveillance policies
18	Eradication of *H. pylori* in patients with established intestinal metaplasia	*H. pylori* management and surveillance policies
19	Biopsy sampling pattern for gastric atrophy / intestinal metaplasia	Histological sampling and pathology infrastructure
20	Handling/labelling of biopsy specimens (separate vs. single container)	Histological sampling and pathology infrastructure
21	Approach to first-degree relatives once GC is diagnosed	*H. pylori* management and surveillance policies
22	Description of gastric atrophy / intestinal metaplasia in the endoscopy report (validated staging scale vs. free text)	Histological sampling and pathology infrastructure
23	Access to an expert gastrointestinal pathologist	Histological sampling and pathology infrastructure
24	Surveillance planning for patients with gastric atrophy / intestinal metaplasia	*H. pylori* management and surveillance policies

No.	Questionnaire item	Domain
25	Frequency of referral of suspicious lesions for high-quality endoscopy	*H. pylori* management and surveillance policies
26	Prescription of aspirin in patients with premalignant gastric conditions	Chemoprevention (contextual item)
27	EGD follow-up of patients with autoimmune gastritis for GC and gastric carcinoids	*H. pylori* management and surveillance policies
28	Access to endoscopic submucosal dissection (locally or after referral)	Therapeutic resources (contextual item)

GC, gastric cancer; EGD, esophagogastroduodenoscopy; UGI, upper gastrointestinal; WLE, white-light endoscopy; HD, high-definition; VCE, virtual chromoendoscopy; PPI, proton-pump inhibitor; OLGA, Operative Link on Gastritis Assessment; OLGIM, Operative Link on Gastric Intestinal Metaplasia; EGGIM, Endoscopic Grading of Gastric Intestinal Metaplasia; MAPS, Management of Epithelial Precancerous Conditions and Lesions in the Stomach.

Supplementary Item 3. Quality indicators included in the composite guideline adherence score used for cluster analysis

Variable	Parameter included in the model	Definition
Endoscopic equipment	Access to high-definition endoscopy with virtual chromoendoscopy (HD-VCE)	Availability and routine use of HD-VCE
Systematic assessment	Use of VCE in every EGD	Routine use of VCE to increase the rate of detection
Endoscopic documentation	Photo-documentation of recommended anatomical stations	Consistent documentation of all recommended gastric landmarks during endoscopy
Reporting quality	Use of structured reporting systems	Utilization of validated scoring systems for gastric atrophy or intestinal metaplasia (e.g., Kimura-Takemoto)
Histological sampling	Adherence to the Sydney biopsy protocol	Standardized biopsy sampling according to the Sydney system
Surveillance	Guideline-based surveillance strategy	Surveillance intervals consistent with international guidelines (e.g. MAPS III)
